# Exome Sequencing of Germline DNA from Non-BRCA1/2 Familial Breast Cancer Cases Selected on the Basis of aCGH Tumor Profiling

**DOI:** 10.1371/journal.pone.0055734

**Published:** 2013-01-31

**Authors:** Florentine S. Hilbers, Caro M. Meijers, Jeroen F. J. Laros, Michiel van Galen, Nicoline Hoogerbrugge, Hans F. A. Vasen, Petra M. Nederlof, Juul T. Wijnen, Christi J. van Asperen, Peter Devilee

**Affiliations:** 1 Department of Human Genetics, Leiden University Medical Centre, Leiden, The Netherlands; 2 Leiden Genome Technology Center, Leiden University Medical Centre, Leiden, The Netherlands; 3 Department of Human Genetics, Radboud University Nijmegen Medical Centre, Nijmegen, The Netherlands; 4 The Netherlands Foundation for the Detection of Hereditary Tumors, Leiden, The Netherlands; 5 Department of Pathology, The Netherlands Cancer Institute, Amsterdam, The Netherlands; 6 Department of Clinical Genetics, Leiden University Medical Centre, Leiden, The Netherlands; IFOM, Fondazione Istituto FIRC di Oncologia Molecolare, Italy

## Abstract

The bulk of familial breast cancer risk (∼70%) cannot be explained by mutations in the known predisposition genes, primarily *BRCA1* and *BRCA2*. Underlying genetic heterogeneity in these cases is the probable explanation for the failure of all attempts to identify further high-risk alleles. While exome sequencing of non-BRCA1/2 breast cancer cases is a promising strategy to detect new high-risk genes, rational approaches to the rigorous pre-selection of cases are needed to reduce heterogeneity. We selected six families in which the tumours of multiple cases showed a specific genomic profile on array comparative genomic hybridization (aCGH). Linkage analysis in these families revealed a region on chromosome 4 with a LOD score of 2.49 under homogeneity. We then analysed the germline DNA of two patients from each family using exome sequencing. Initially focusing on the linkage region, no potentially pathogenic variants could be identified in more than one family. Variants outside the linkage region were then analysed, and we detected multiple possibly pathogenic variants in genes that encode DNA integrity maintenance proteins. However, further analysis led to the rejection of all variants due to poor co-segregation or a relatively high allele frequency in a control population. We concluded that using CGH results to focus on a sub-set of families for sequencing analysis did not enable us to identify a common genetic change responsible for the aggregation of breast cancer in these families. Our data also support the emerging view that non-BRCA1/2 hereditary breast cancer families have a very heterogeneous genetic basis.

## Introduction

The genetic landscape of breast cancer susceptibility known to date is constituted by more than 30 gene loci. Mutations in some of these, like BRCA1 and BRCA2, are extremely rare, but confer high risks to breast cancer, others are common but only confer a minor increase in risk. However, jointly these alleles explain less than 30% of the familial breast cancer risk [1]–[3]. When considering families with multiple cases of early-onset breast cancer in which mutations in the known high-risk genes have been excluded (hereafter: “BRCAX” families), an unknown, rare, highly penetrant allele would appear to be the most parsimonious genetic explanation. However, linkage studies have not discovered any major breast cancer susceptibility gene since the identification of *BRCA1* and *BRCA2*. This suggests that these high-risk alleles are too rare to be detected by linkage studies in unselected BRCAX families.

Therefore, an important factor determining the success of a genome-wide search for linkage in a set of BRCAX families is the extent of underlying genetic heterogeneity. Simulation studies have shown that study power drops sharply if mutations in the sought-after new gene explain <30% of the investigated families. Selecting families based on a shared phenotype might lead to a genetically more homogeneous group of families, which are more likely to share variants in the same gene. A shared phenotype might be defined by the presence of certain cancer types in the family. For example, linkage analysis of non-BRCA1 breast cancer families with a case of male breast cancer, led to the discovery of the *BRCA2* locus [4]. Also, certain histopathological features of tumours might be used to identify subgroups. It has been shown that breast tumours from BRCA1 and BRCA2 mutation carriers show specific genomic profiles as determined by comparative genomic hybridization (CGH) [5]–[10].

We recently described a specific array comparative genomic hybridization (aCGH) profile in a subgroup of BRCAX breast tumours [11]. This aCGH-profile is characterized by a gain of almost whole chromosome 22, in combination with some other specific changes, and was observed to be present in multiple breast cancer cases contained within six of the 27 analyzed BRCAX families. We hypothesized that these six families might have mutations in the same high-risk breast cancer gene. Here we present linkage analysis of these six families as well as exome sequencing of two family-members from each.

## Methods

### Patients

**Figure 1:**
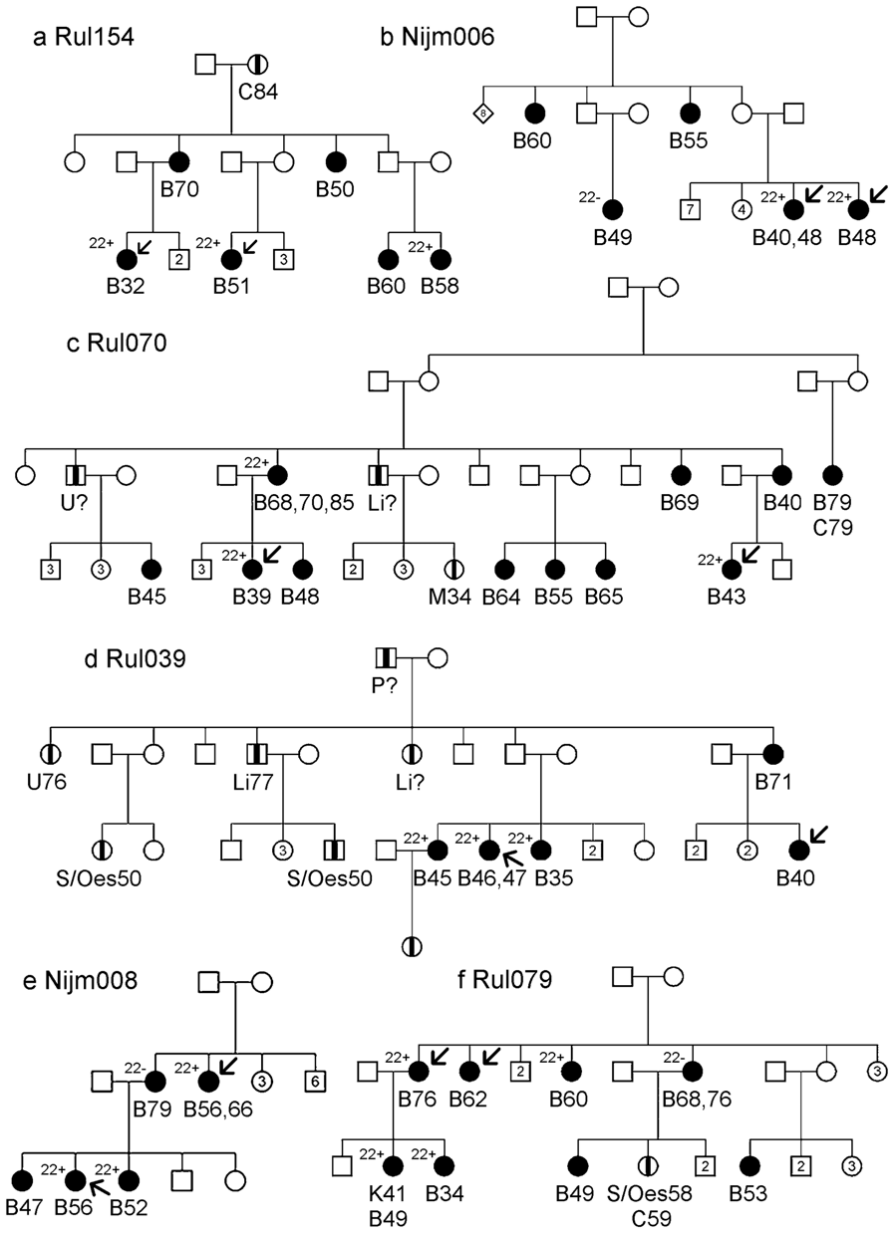
Pedigrees of the families in which multiple tumours showed the “22-gain-like” aCGH profile. Individuals affected with breast cancer are represented by a filled square or circle. Individuals affected by another type of cancer are represented by a square or circle with a vertical black stripe. Below the age at diagnosis and type of cancer can be found: B stands for breast cancer, Li or liver cancer, S for stomach cancer, Oes for oesophagus cancer, C for colon cancer, M for melanoma, Cvx for Cervix cancer, K for kidney cancer, P for prostate cancer and U for type of cancer unknown. Arrows point at the individuals at whose DNA was used for exome sequence. Individuals with tumours with and without the “22-gain-like profile” are represented by “22+”and “22−”.

Previously, we determined the aCGH profiles of 58 breast tumours from 27 BRCAx families. A detailed description of the original selection criteria of the BRCAx families is given in Didraga et al. [11]. We selected six of these families in which the tumours of multiple cases showed the 22-gain-like profile. The pedigrees of these families are depicted in Figure 1a-f. The occurrence of cancer was assessed through the index case and whenever possible verified with pathology reports. The number of breast cancer cases per family ranged from five to eleven, with a mean age of onset of 54 years. No male breast cancer cases and no ovarian cancer cases were reported. In total 46 breast tumours were diagnosed in these families, of which four were second primary tumours. One breast cancer case developed a kidney tumour and another breast cancer case was diagnosed with colon cancer. Other cancers that occurred in these families were liver cancer (n = 3), stomach/oesophagus cancer (n = 3), colon cancer (n = 2), melanoma (n = 1), cervical cancer (n = 1), prostate cancer (n = 1) and two cancers of unknown type. All participants provided written informed consent and approval of the medical ethical committee at the Leiden University Medical Centre was obtained.

### Linkage Analysis

The six selected families are part of a larger cohort of  = 55 families, which were genotyped before by Oldenburg et al [12] for a genome-wide linkage analysis study. In brief, all individuals from whom DNA was available were genotyped using the Linkage Mapping Set MD10 from Applied Biosystems consisting of 400 markers which results in a 10 centimorgan resolution. Genotypes were called automatically using Genemapper software(Applied Biosystems) and checked manually. Allele frequencies were calculated based on one randomly chosen individual from each family. The UNKNOWN program of the LINKAGE package [13] was used to check for Mendelian errors. If after manual reassessment of the raw data Mendelian errors could not be solved these genotypes were changed to “untyped” (i.e., “0 0”). We performed a multipoint linkage analysis using Genehunter software (version 2.1 B) [14]. We assumed a model with a dominant susceptibility allele with an allele frequency of 0.003. Breast cancer risk at age 80 for carriers of the risk allele was assumed to be 0.85. For non-carriers we assumed a risk of 0.096. Risks were modelled in seven age categories as described by Easton et al. [15]. Under the assumption of homogeneity, the LOD scores of the six families linked to the 22-gain profile were added up. To define the limits of a linkage region we took the maximum LOD score minus one as a cut-off.

### Exome Sequencing

Genomic DNA was extracted from peripheral blood using standard protocols. Samples were prepared according to the manufacturers protocol (SureSelect All Exon (v1), Agilent Technologies) with some minor adjustments. In brief, for each individual 5 µg DNA was fragmented using adaptive focused acoustics (Covaris S-series single tube) in order to get fragments of 200–300 bp. Primer oligonucleotides for paired-end sequencing (Illumina) were ligated to both ends of the fragment. Of each sample 500 ng was then hybridized with 2.5 µl SureSelect Oligo Capture Library for 20 hours. After multiple washing steps, the captured DNA was amplified in order to get sufficient DNA for the sequencing experiment. Paired-end flow cells were then prepared on a cluster station according to the manufactures protocol (Illumina), using one lane per sample. Sequencing was the performed on a Genome Analyzer IIx (Illumina) with a paired-end module, generating 75 base pair reads.

### Data Analysis

Alignment of the reads was done using the GAPPSv3 pipeline. Before alignment raw reads were filtered for adapter sequences and low quality bases using the FastxToolkit [16]. Alignment to the human reference genome (hg19, GRCh37) was done using Stampy [17] which integrates BWA [18] for bulk alignment and its own algorithm for complex regions. For detailed settings see Table S1. Variants were called with VarScan [19]. Filter settings applied a minimum coverage of 10 times at the variant position, and a variant allele frequency of at least 30% of the reads. In the region of the linkage peak we increased the sensitivity by calling variants if the variant allele was supported by at least 15% of the reads. Annotation of the variants was done using SeattleSeq (version 7.01, [20]). Assuming that causal variants are rare, we removed all variants with an allele frequency >1% in either HapMap [21], 1000 genomes (phase 1) [22], exome variant server (v.0.0.11, ESP5400, [23]) or our in-house variant database (containing 298 non-cancer exomes). In addition, variants that were found in a homozygous state in at least one of the twelve individuals were removed.

### Sanger sequencing and melting curve analysis (MCA)

Validation of variants was done using PCR following standard protocols, followed by Sanger sequencing on an ABI3730XL sequencer. To assess variant frequencies in familial breast cancer cases and controls, high resolution melting curve analysis was performed. Non-BRCA1/2 familial breast cancer cases (n = 531) were obtained from the clinical genetics centre Leiden and healthy controls (n = 458) were obtained from the Dutch blood bank, Sanquin. PCR was performed in a 1∶10∶10 forward primer: reverse primer: probe ratio in the presence of LC green (Idaho Technology Inc.). Melting curves were assessed on a LightScanner (Idaho Technology Inc.) for temperatures between 50°C and 90°C and analyzed with Call-IT software (Idaho Technology Inc.). All primer and probe sequences are available upon request.

## Results

**Figure 2:**
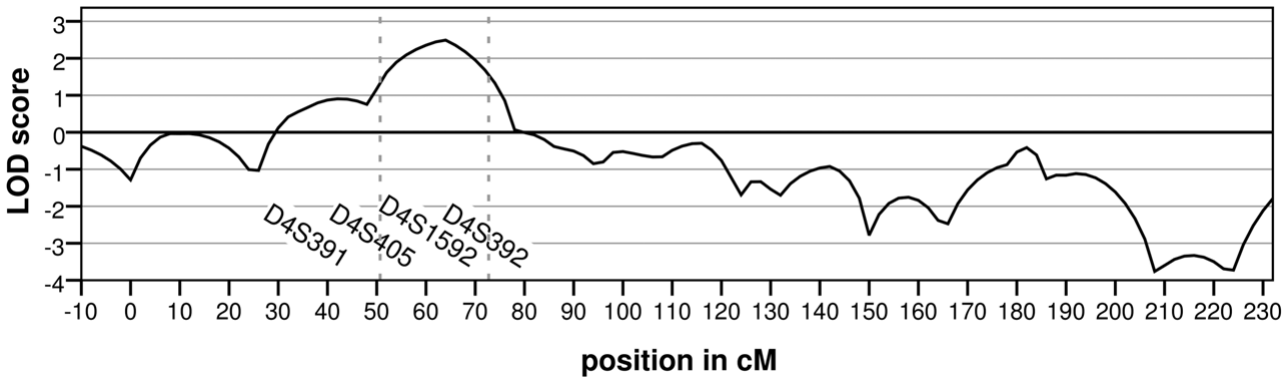
Linkage on chromosome 4 for the families in which multiple tumours showed the “22-gain-like” aCGH profile. The LOD-score was calculated under the assumption of homogeneity. The dashed lines indicate the maximum LOD-score -1interval. The X-axis shows the position on chromosome 4 in centimorgan and the markers with a LOD score >0 are indicated. The highest LOD score of 2.49 was located at marker D4S405.

We previously analysed the breast tumours of 58 patients from 27 BRCAX families using aCGH [11]. Hierarchical clustering identified several subgroups of BRCAX tumours, one of which was characterized by a gain of chromosome 22. Remarkably, in 6 families, tumours from multiple patients displayed this chromosome 22 gain profile (Figure 1). Linkage analysis under homogeneity revealed a linkage peak with a LOD score of 2.49 on chromosome 4 in these six families (Figure 2 and Figure S1). The next highest linkage peak was 1.04 at 10q and no other linkage peaks with a LOD score greater than 1.0 were detected.

A 25-Mb candidate region (chr4:40.000.000-65.000.000) was defined as the region showing a LOD score greater than the peak LOD score minus one. Two individuals per family were selected for exome sequencing, usually at least second-degree relatives (figure 1). (Details on coverage of the individual exomes can be found in Figure S2 and S3.) This revealed on average 499 variants in the candidate region that were shared by both individuals of a family. After removing intergenic and non-conserved variants in non-coding regions, five variants remained (Table 1). However, none of the genes carrying these variants were found to do so in two or more families. Hence mutations in a single gene are less likely to explain the linkage result. We then considered the possibility that two or more genes in the chromosome 4 region each fortuitously carries a high-risk mutation in one of the six families. Of the detected variants, three synonymous variants in three genes (*FRYL*, *AASDH*, *PPAT*) were not further examined, because these variants are unlikely to affect protein function. A missense variant in *REST* and a well-conserved 3′UTR variant in *LNX1* were validated by Sanger sequencing. The *LNX1* variant was present in five of eight cases in family RUL070. The missense variant in *REST* was detected in six out of seven cases in family RUL079, however Grantham and conservation scores for this variant were low (Grantham = 45, Phastcons = 0.00, GERP = −3.56) and Polyphen [24] predicts it to be benign.

**Table 1:** Well conserved or coding variants in the linkage region on chromosome 4.

Variant	Gene	Family	Effect	rs-number	PhastCons^1^	GERP^1^
Chr4:g.48545947T>C	FRYL	RUL070	Synonymous	-	1.00	1.33
Chr4:g.54327036_54327037insATT	LNX1	RUL070	3′ UTR	57366823	0.97	4.56
Chr4:g.57248742A>C	AASDH	RUL070	Synonymous	146114987	1.00	−0.43
Chr4:g.57261623G>A	PPAT	RUL070	Synonymous	-	0.22	−5.98
Chr4:g.57797037G>T	REST	RUL079	Missense^2^	138787075	0.00	−3.56

1 Phastcons and GERP are both regional conservation algorithms ranging from 0 to 1 and −12.3 to 6.17 respectively (1 and 6.17 being most conserved).

2 Grantham = 45, PolyPhen prediction = Benign.

Finally, we examined the possibility that the six families shared variants in a gene outside the linkage peak region (whole exome). We first focused on variants that were likely to result in a truncated protein (gained stop-codon, frameshift and splice-site variants). In the six families we found in total 49 different, rare protein-truncating variants in 48 genes. A number of genes showed a protein-truncating variant shared by several families. However, all these variants were present in regions whose sequences showed large similarities with regions elsewhere in the genome. When examining the unprocessed sequence-reads of the families in which the variants were not called, in most instances the variant could be detected, but in fewer reads than the required threshold of 30%. Thus, we considered all these variants to be false-positive findings resulting from sequence read-mapping errors. Indeed, the only one of these variants that we followed up by Sanger sequencing was a splice-site mutation in *FANCD2*. *FANCD2* is a Fanconi Anaemia gene and therefore a candidate breast cancer gene. However, upon re-sequencing, this variant was not present in *FANCD2*, but in a region with a similar sequence elsewhere on chromosome 3 near *EMC3* (data not shown).

After removing the variants resulting from read-mapping errors, 21 truncating variants remained (see table S2). All were present in only one of the six families. Of these variants a frameshift mutation, c.811delT, in *HAUS3* was potentially interesting, because *HAUS3* has been reported to be somatically mutated in a lobular breast tumour [18]. Sanger sequencing showed that five out of seven breast cancer patients in RUL079 had this deletion. High resolution melting curve analysis of this specific variant did not reveal any additional carriers among 531 familial breast cancer cases. However, three individuals in a group of 458 healthy controls were found to carry the c.811delT, dismissing it as a high-risk breast cancer allele.

We also took into account possibly damaging missense variants. This was defined as missense variants with either a Grantham score >100, a GERP conservation score >3, a PhastCons conservation score >0.7 or a “probably damaging” PolyPhen2 prediction. Due to the large number of variants remaining (n = 657), following up all variants with Sanger sequencing was deemed impractical. We therefore selected variants with a function in DNA integrity maintenance, because the majority of breast cancer susceptibility genes identified to date have a function in this pathway (table 2). Again, no genes were found to have a variant in more than one family. However, some individual families showed possibly damaging variants in genes (n = 8) involved DNA damage repair or chromosome segregation, shared by both assayed individuals. One of these variants, present in *RBMX*, could not be validated. However, a variant in *HLTF*, p.S378T, was present in five out of five cases of family NIJM008. This variant was selected because of a high GERP conservation score (3.15). The PhastCons conservations score, however, was only 0.21 and this variant was predicted to be benign by Polyphen2. Sanger sequencing showed that the remaining six variants, in *CASC5*, *CUL9*, *MUTYH*, *SMC6*, *TTK* and *XRCC2*, had a poor or moderate co-segregation with disease (Figure S4). Interestingly, the variant in XRCC2 was also detected in an Australian family and therefore further analysed in an international mutation scanning effort [25]. A significant association between rare XRCC2 variants and familial breast cancer was reported. However, a large validation study was not able to confirm this association [26].

**Table 2:** Possibly damaging or well conserved variants in genes encoding proteins involved in DNA integrity maintaince.

Gene	Variant	Grantham	GERP^1^	PhastCons^1^	PolyPhen2	Function
CASC5	p.I26L	5	4.53	0.999	Probably damaging	Spindle-assembly checkpoint signaling and chromosome alignment
CUL9	p.S2328F	155	5.03	0.989	Possibly damaging	Regulates the subcellular localization of p53 and subsequent function
HLTF	p.S378T	58	3.15	0.208	Benign	Error-free postreplication repair of damaged DNA
MUTYH	p.I223V	29	5.43	1	Benign	Oxidative DNA damage repair
RBMX	p.Y357H	83	5.66	1	Probably damaging	Regulation of programmed cell death in breast cancer and homologous recombination
SMC6	p.R403W	101	2.65	0.998	Probably damaging	DNA damage repair via homologous recombination
TTK	p.R185W	101	4.04	0.004	Probably damaging	Chromosome alignment, centrosome duplication and critical mitotic checkpoint
XRCC2	p.R91W	101	4.48	0.742	Probably damaging	DNA damage repair via homologous recombination

Variants were selected if either of these criteria was met: Grantham score>100, GERP conservation score>3, PhastCons conservation score>0.7, or a “Probably damaging” Polyphen2 prediction.

1 Phastcons and GERP are both regional conservation algorithms ranging from 0 to 1 and −12.3 to 6.17 respectively (1 and 6.17 being most conserved).

## Discussion

The landscape of genetic risk factors for breast cancer is known to be diverse, ranging from rare high-risk alleles, like *BRCA1* and *BRCA2*, to common polymorphisms that only confer a minor breast cancer risk increase. The large proportion of familial breast cancer cases that is not explained by the genetic risk factors known to date are thought to have a very heterogeneous basis [1]–[3]. Both segregation analysis [27]–[29] and the fact that no major high-risk breast cancer genes have been identified since *BRCA2* suggest that additional high-risk alleles are much rarer than mutations in *BRCA1* and *BRCA2*. Exome sequencing might be a very useful tool to identify these very rare high-risk alleles. However, finding novel disease alleles among thousands of not-pathogenic variants might be more complex in a common and genetically heterogeneous disease like breast cancer, than in the rare Mendelian phenotypes in which exome sequencing has been very successful to date [30]. Therefore selecting a genetically more homogeneous patient subgroup seems crucial.

We hypothesized that by selecting BRCAX families with a similar phenotype, we would enrich our study population for families with germline mutations in the same gene. In this study six BRCAX families in which the majority of tumours show a previously identified aCGH profile [11] were selected. Linkage analysis in these families showed a peak on chromosome 4, which suggested that these families might share a genetic aetiology. Massively parallel sequencing after whole-exome capture was performed on two individuals per family, but no genes were identified in which more than one family showed a likely pathogenic variant after assessing the predicted effect on the protein and co-segregation. Nonetheless, we did detect multiple possibly pathogenic variants in genes that encode for DNA integrity maintenance proteins outside the linkage peak region. However, none remained as likely causes of familial clustering of breast cancer because of poor co-segregation or relative high frequency of the specific variant in a control population.

It is important to realize that, by enriching our samples for the coding regions of the DNA, we might have missed relevant variants in the promoter, deep intronic regions affecting splicing or in regulatory regions further away from the causal gene. However, such mutations seem to represent only a minority of the mutation mechanisms in the known disease-related genes, as recorded in OMIM and other public databases [31]. It seems less likely therefore, that all families in our study population were due to such mutations. In addition, variants outside the coding regions are much harder to interpret functionally, and a whole-genome sequencing approach would have resulted in thousands of variants of uncertain clinical significance.

Multiple studies have shown that aCGH classifiers can be built to distinguish *BRCA1* and *BRCA2* tumours from sporadic tumours and each other [5]–[10]. These studies suggest that tumours of patients with mutations in the same gene also share a somatic genetic aetiology. Alvarez [32] and colleagues found that part of the BRCAX tumours showed aCGH profiles similar to those of *BRCA1* tumours. A large proportion of these tumours turned out to have hypermethylation of *BRCA1*. Some studies that performed aCGH profiling on BRCAX tumours find similarities with profiles of *BRCA2* tumours [33], [34], suggesting that either a cause of *BRCA2* inactivation in these tumours has yet to be detected or that inactivation of a number of genes can lead to a similar aCGH profile. It might be that patients with the 22-gain profile do not share mutations in the same gene, but in the same pathway. In order to detect an enrichment of deleterious variants in a specific pathway, a large number of familial patients with 22-gain tumours will need to be sequenced, preferably in conjunction with gene expression profiling of tumours; however it will be challenging to collect sufficient numbers samples for such an effort.

Another possibility is that patients with a 22-gain tumour have mutations in a moderate risk gene.

Muranen et al. [35] have shown that specific aCGH features occur significantly more often in tumours of patients with a CHEK2*1100delC mutation. This suggests that also moderate risk germline mutations can lead to a homogenous phenotype. By only assessing variants that are shared by both family members and discarding variants that show poor co-segregation, we may have missed variants in a moderate risk gene. In addition, moderate risk variants might have an allele frequency of more than 1% as has been shown to be true for the CHEK2*1100delC mutation in some populations [36]. However without using these selection criteria, it would not have been possible to limit possibly interesting variants to a number that is manageable to follow-up. Therefore a study design that includes exome sequencing in a very limited number of familial cases is underpowered to detect moderate risk variants.

A good balance between stringent selection criteria (to limit the number of variants for follow-up) and not excluding too many potentially interesting variants is difficult to find. An excess of rare genetic variants due to recent explosive growth of the human population has been observed [37], [38]. This makes it difficult to interpret the effect of a very rare variant on breast cancer risk outside the family it was originally detected in. For example, the missense variant we detected in XRCC2 was also found in an Australian BRCAX family [25]. Whereas we had initially dismissed this variant because it did not show convincing co-segregation with disease, the fact that Park et al. had also found a protein-truncation variant in XRCC2, prompted a mutation scan of a large population of familial breast cancer cases and controls. This detected a significant association between familial breast cancer and XRCC2 [25]. However, an even larger international validation of these results was unable to confirm this association [26]. This leaves the possibility that some very rare XRCC2 alleles are true breast cancer susceptibility alleles, but conferring only moderate risks, which would require huge association studies to demonstrate. This example emphasizes the importance of international collaboration and sharing of data, both in the variant selection and in the validation phase.

In conclusion, we did not find evidence for mutations in a rare high-risk gene in a subgroup of BRCAX cases defined by an aCGH profile. Although, we cannot rule out that these families have mutations in genes belonging to the same pathway or in a non-coding region. Exome sequencing efforts in large cohorts of BRCAX cases are needed to definitively unravel the genetic basis underlying the aetiology of unexplained familial clustering of breast cancer and its link with tumour characteristics.

## Supporting Information

Figure S1 **Parametric LOD scores of the individual families in the linkage region on chromosome 4.** The X-axis shows the position on chromosome 4 in centimorgan.
(TIF)

Figure S2 **Percentage of CCDS exon bases covered at least 10× per individual.** CCDS = consensus coding sequence.
(TIF)

Figure S3 **Mean coverage of CCDS exons per individual.** CCDS = consensus coding sequence.
(TIF)

Figure S4 **Segregation of selected variants within the families (a–d).** Individuals carrying or not carrying a specific variant are indicated with a “+” or with a “−”respectively. The p.Y357H variant in RBMX, which was detected by massive parallel sequencing in family Nijm006, could not be validated by Sanger sequencing.
(TIF)

Table S1 Description of the data analysis settings. Software versions used in the data analysis including details on settings.
(DOC)

Table S2 Truncating variants all detected in only one of the six families. Truncating variants with an allele frequency <1% in HapMap [21], 1000 genomes (phase 1) [22], exome variant server (v.0.0.11, ESP5400, [23]) and our in-house variant database. All variants were present in only one of the six families. * Splice site affected at position c.2418+2 ** Splice site affected at position c.982-1
(DOC)
