# Supplementary figures and images for: Exome Sequencing of Germline DNA from Non-BRCA1/2 Familial Breast Cancer Cases Selected on the Basis of aCGH Tumor Profiling
Source: PLoS One. 2013 Jan 31;8(1):e55734. doi: 10.1371/journal.pone.0055734 (PMC3561352; doi:10.1371/journal.pone.0055734)

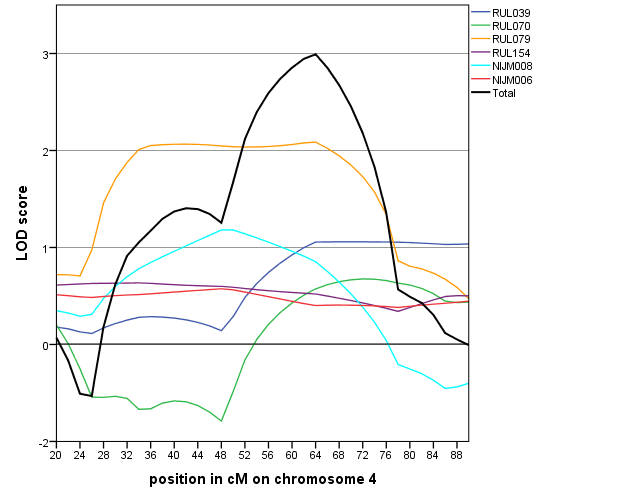

Supplement: Figure S1 — Parametric LOD scores of the individual families in the linkage region on chromosome 4. The X-axis shows the position on chromosome 4 in centimorgan. (TIF) [file pone.0055734.s001.tif]

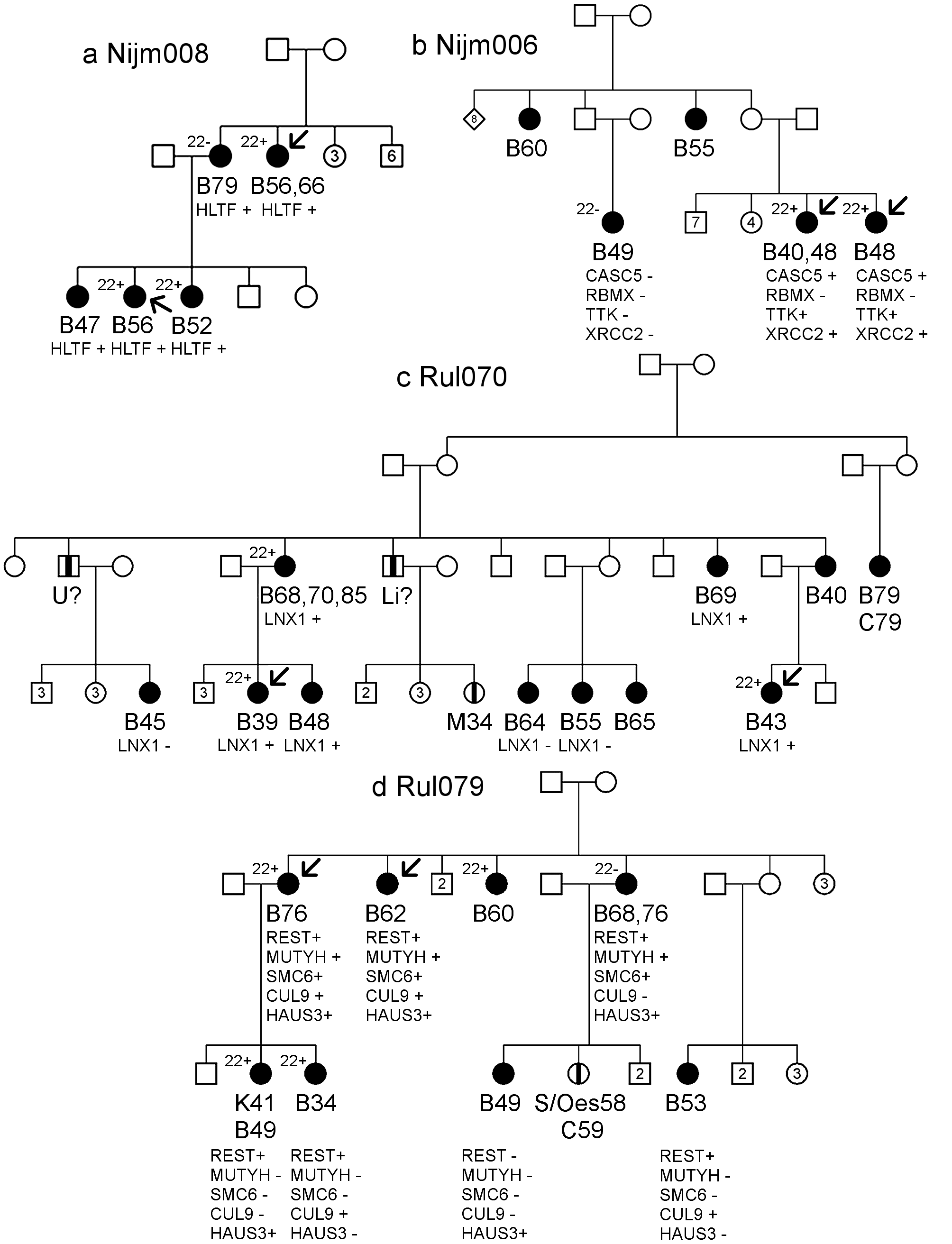

Supplement: Figure S4 — Segregation of selected variants within the families (a–d). Individuals carrying or not carrying a specific variant are indicated with a “+” or with a “−”respectively. The p.Y357H variant in RBMX, which was detected by massive parallel sequencing in family Nijm006, could not be validated by Sanger sequencing. (TIF) [file pone.0055734.s004.tif]
