# Supplementary material for: Exome Sequencing of Germline DNA from Non-BRCA1/2 Familial Breast Cancer Cases Selected on the Basis of aCGH Tumor Profiling
Source: PLoS One. 2013 Jan 31;8(1):e55734. doi: 10.1371/journal.pone.0055734 (PMC3561352; doi:10.1371/journal.pone.0055734)
Supplement: Table S1 — Description of the data analysis settings. Software versions used in the data analysis including details on settings. (DOC) [file pone.0055734.s005.doc]

**Table S1** Description of the data analysis settings

| **Software** | **Version** | **Settings** |
| --- | --- | --- |
| FastxToolkit | 0.0.13 | minimum base quality=20  minimum percentage base with minimum quality=80  minimum read length after clipper/trimmer=25 |
| FastQC | v0.8.0 | Default |
| Stampy | v1.0.12 | Default |
| BWA | 0.5.6 | Default |
| Samtools | 0.1.14 | Default |
| VarScan | v2.2 | See material and methods section |
