# Supplementary material for: Exome Sequencing of Germline DNA from Non-BRCA1/2 Familial Breast Cancer Cases Selected on the Basis of aCGH Tumor Profiling
Source: PLoS One. 2013 Jan 31;8(1):e55734. doi: 10.1371/journal.pone.0055734 (PMC3561352; doi:10.1371/journal.pone.0055734)
Supplement: Table S2 — Truncating variants all detected in only one of the six families. Truncating variants with an allele frequency <1% in HapMap [21], 1000 genomes (phase 1) [22], exome variant server (v.0.0.11, ESP5400, [23]) and our in-house variant database. All variants were present in only one of the six families. * Splice site affected at position c.2418+2 ** Splice site affected at position c.982-1 (DOC) [file pone.0055734.s006.doc]

**Table S2** Truncating variants all detected in only one of the six families

| **Gene** | **Variant** | **Function** |
| --- | --- | --- |
| A4GNT | p.R226X | Glycosyltransferase |
| ADAMTS7 | p.N1353fs | Metallopeptidase |
| ANO8 | p.? * | Ca2+-activated Cl− channel |
| CRNKL1 | p.Q50X | Pre-mRNA splicing |
| CPA3 | p. ? ** | Secretory granule metalloexopeptidase |
| HAUS3 | p.C271fs | Microtubule generation within the mitotic spindle |
| HIST1H2BE | p.S7fs | Member of the histone H2B family |
| IFNK | p.W13X | Glycoprotein, important in host defenses against viral infections |
| KCNQ5 | p.Q824X | Potassium channel |
| KIAA1751 | p.A732fs | Unknown |
| LOC100132900 | p.R90X | Unknown |
| MSGN1 | p.Q106X | Mesoderm maturation |
| NIPSNAP3A | p.Q124fs | Putative role in vesicular transport |
| OTOP3 | p.Q82X | Unknown |
| PKD1L2 | p.G235fs | May function as a component of cation channel pores |
| RETNLB | p.I12fs | Important for epithelial barrier function and integrity |
| TAS2R8 | p.F245fs | Taste receptor |
| TBC1D17 | p.F546fs | Unknown |
